# The antimicrobial peptide pardaxin exerts potent anti-tumor activity against canine perianal gland adenoma

**DOI:** 10.18632/oncotarget.2959

**Published:** 2014-12-10

**Authors:** Chieh-Yu Pan, Chao-Nan Lin, Ming-Tang Chiou, Chao Yuan Yu, Jyh-Yih Chen, Chi-Hsien Chien

**Affiliations:** ^1^ Department and Graduate Institute of Aquaculture, National Kaohsiung Marine University, Nanzih Dist., Kaohsiung, Taiwan; ^2^ Graduate Institute and Department of Veterinary Medicine, College of Veterinary Medicine, National Pingtung University of Science and Technology, Neipu, Pingtung, Taiwan; ^3^ Genomics BioSci & Tech Co., Ltd., Xizhi Dist., New Taipei, Taiwan; ^4^ Marine Research Station, Institute of Cellular and Organismic Biology, Academia Sinica, Jiaushi, Ilan, Taiwan

**Keywords:** antimicrobial peptide, pardaxin, cancer treatment, perianal gland adenoma, canine, intratumoral treatment

## Abstract

Pardaxin is an antimicrobial peptide of 33 amino acids, originally isolated from marine fish. We previously demonstrated that pardaxin has anti-tumor activity against murine fibrosarcoma, both *in vitro* and *in vivo*. In this study, we examined the anti-tumor activity, toxicity profile, and maximally-tolerated dose of pardaxin treatment in dogs with different types of refractory tumor. Local injection of pardaxin resulted in a significant reduction of perianal gland adenoma growth between 28 and 38 days post-treatment. Surgical resection of canine histiocytomas revealed large areas of ulceration, suggesting that pardaxin acts like a lytic peptide. Pardaxin treatment was not associated with significant variations in blood biochemical parameters or secretion of immune-related proteins. Our findings indicate that pardaxin has strong therapeutic potential for treating perianal gland adenomas in dogs. These data justify the veterinary application of pardaxin, and also provide invaluable information for veterinary medicine and future human clinical trials.

## INTRODUCTION

For several years, nude mice have been used as *in vivo* experimental models to study the effects of anti-cancer agents. Working with nude mice allows fine control of the experimental conditions *in vivo*, and the resulting findings are thus reliable and reproducible. However, the oncogenic processes in transplanted cancer cells in mouse models and naturally-occurring cancers in humans are not the same, and consequently, differences in tumor development and responses are observed [1]. Therefore, it is important to study cancers that develop naturally in the context of an immune system, as this enables observation of tumor growth over long time periods within a syngeneic host, metastasis to relevant distant sites, the development of recurrence, and the tumor microenvironment [2], all of which may play important roles in cancer treatment. Cancer in dogs shares many features with human cancer, including histological morphology, molecular targets, and molecular mechanisms; furthermore, current therapies for human and dog cancer are similar [3, 4]. Several investigations have developed novel treatment options for cancer (including urinary bladder cancer [5], canine osteosarcoma [6], B-cell lymphoma [7], prostatic hyperplasia [8], and solid tumor [9]) using dogs as models, thus emphasizing the importance of studying naturally-occurring cancers in dogs for gaining insights into the biology of human cancer.

We previously reported that intratumoral injection of certain antimicrobial peptides (AMPs) induces anti-tumor responses [10, 11]. The intratumoral injection technique has undergone extensive development, and now enables direct application of drugs to the tumor microenvironment, thereby reducing tumor size [12-14]. Following intratumoral injection, AMPs may engage in electrostatic interactions with the cell membrane, thereby bringing about membrane disruption and rapid necrotic cell death [12, 15-17]. Other studies reported that AMPs not only trigger necrosis through cell membrane lysis, but also activate apoptosis in cancer cells via the mitochondrial lytic effect in the presence of anionic lipids [18-20].

Pardaxin is an AMP originally isolated from a marine fish species (*Pardachirus marmoratus*); pardaxin has been shown to permeabilize the virion membrane, perturb model membranes of phosphatidyl choline and serine, stimulate calcium uptake in PC12 cells, exhibit ionic channel selectivity, and exert antibacterial and anti-tumor activity [10, 21-25]. Synthetic pardaxin peptide inhibits the proliferation of HT1080 cells in a dose-dependent manner, and induces programmed cell death in HeLa cells [26]. Proteomic analysis revealed that pardaxin triggers apoptotic signaling pathways in HeLa cells, which undergo cross-talk with the unfolded protein response (UPR), the c-Jun pathway, and reactive oxygen species (ROS). Pardaxin plays important roles in the scavenging of ROS to alleviate c-Jun activation; small interfering RNA-mediated knockdown of c-Jun abrogates pardaxin-induced caspase activation and cell death, thereby implicating ROS and c-Jun in pardaxin-induced apoptosis signaling [27]. Transcriptome analysis of pardaxin-treated HT-1080 cells revealed induction of the gene encoding c-FOS, an AP-1 transcription factor. Treatment with pardaxin increased cellular levels of calcium, and blockage of cellular calcium signaling disrupts pardaxin-induced cell death [28]. Injection of pardaxin into the intratumoral space in mice significantly inhibited tumor growth [10]. However, the anti-tumor effects of pardaxin, and their underlying mechanisms, have not been studied in non-murine animal models. In order to elucidate the mode of action of pardaxin in more detail, we performed a pilot study on the anticancer activities of pardaxin administered to canine tumors.

We evaluated the efficacy of pardaxin as an anticancer drug *in vivo* in a total of 14 dogs with naturally-occurring cancer (nine with mammary gland tumors, one with fibrocarcinoma, two with squamous cell carcinoma, four with perianal gland adenoma, two with histiocytoma, three with malignant mast cell tumor, and one with sarcoma). In addition, the effect of pardaxin on tumor size, immune-related gene expression, and blood biochemical parameters were delineated.

## RESULTS

### Characteristics of dogs enrolled in this study

The dog patient demographics and tumor types are listed in Table 1. A total of 14 dogs were enrolled into the dose escalation portion of the study. The patient population consisted of dogs of various breeds: mixed breed (n = 7), German Shepherd Dog (n = 1), Golden Retriever (n = 1), Maltese (n = 1), Yorkshire Terrier (n = 1), American Pit Bull Terrier (n = 1), Shih Tzu (n = 1), and Husky (n = 1). The following tumor types were included in the study: mammary gland tumor (n = 9), fibrocarcinoma (n = 1), squamous cell carcinoma (n = 2), perianal gland adenoma (n = 4), histiocytoma (n = 2), malignant mast cell tumor (n = 3), and sarcoma (n = 1).

**Table 1:** Properties of dogs enrolled in this study

Number	Medical record numbers	Sex	Age	Breed	Tumor
1	02-13-26	♀	7Y	German Shepherd Dog	Mammary gland tumor
2	02-15-02	♀	20Y	Mix	Mammary gland tumor
3	01-85-03	♀	4-5Y	Golden Retriever	Fibrosarcoma
4	02-14-61	♀	11Y	Mix	Squamous Cell Carcinoma
5	02-09-26	♀	10Y	Maltese	Mammary gland tumor
6	02-15-91	♂	8Y	Mix	Perianal gland adenoma
7	02-16-25	♂	3-4Y	Mix	Histiocytoma
8	02-16-06	♀	14Y	Yorkshire Terrier	Mammary gland tumor
9	02-21-80	♀	7Y	Mix	Malignant Mast Cell Tumor
10	02-22-62	♂	2Y	American Pit Bull Terrier	Histiocytoma
11	02-21-80	♀	7Y	Mix	Malignant Mast Cell Tumor
12	01-70-90-4	♀	9Y	Mix	Sarcoma
13	HW4989	♂	9Y	Shih Tzu	Perianal gland adenoma
14	02-29-90	♂	9Y	Husky	Perianal gland adenoma

### Drug administration and anti-tumor activity in tumor-bearing dogs

**Figure 1:**
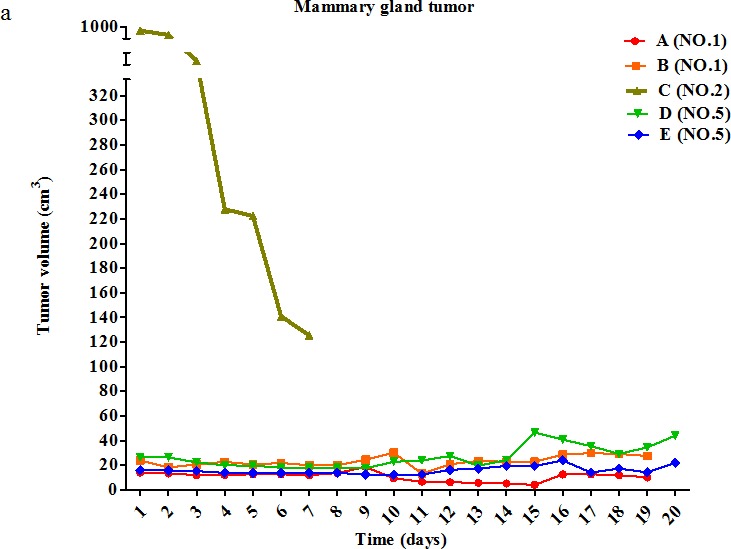

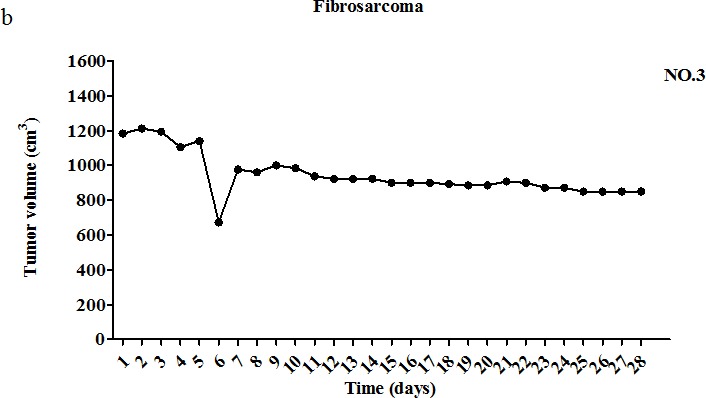

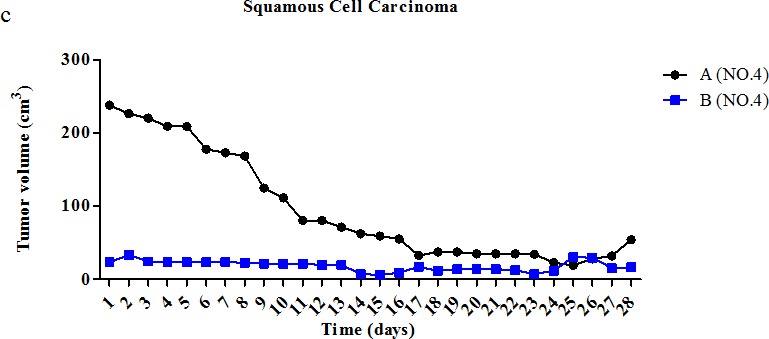

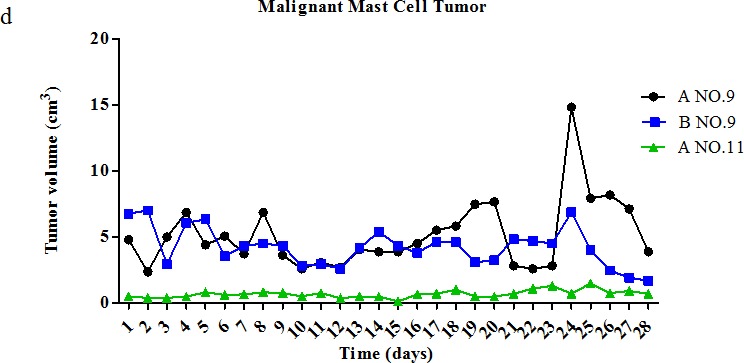

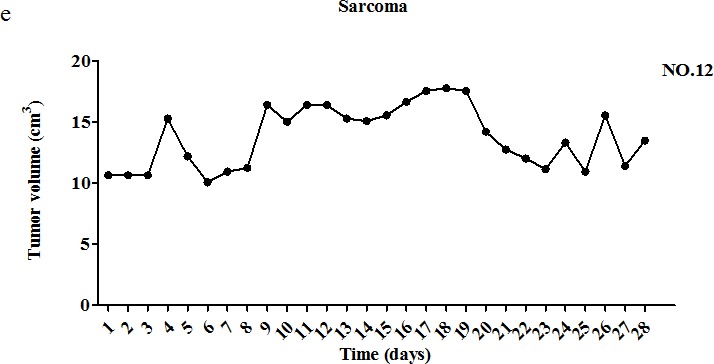
Change in tumor volume during treatment with pardaxin (a) Five mammary tumors (of dogs #1, #2, and #5) were monitored for 20 days. (b) One fibrosarcoma tumor (of dog #3) was monitored for 28 days. (c) Two squamous cell carcinomas (of dog #4) were monitored for 28 days. (d) Three malignant mast cell tumors (of dogs #9 and #11) were monitored for 28 days. (e) One sarcoma tumor (of dog #12) was monitored for 28 days. Descriptions of tumor features and pardaxin treatment regimens are provided in Tables 1 and 2. The first day of pardaxin administration is labeled as day one. Each tumor of each dog was injected with a different concentration of pardaxin, and results are plotted using different colors. The tumor volumes (cm^3^) of individual tumors are shown.

Dog patients with various tumor types were given daily intratumoral injections of pardaxin at dosages ranging from 0.010 mg/cm^2^ to 16.666 mg/cm^2^ (Table 2). Dog patients #1, #2, #5, and #8 had mammary gland tumors; while the volume of the tumor in dog #2 decreased during pardaxin treatment, the growth of the other mammary gland tumors were not substantially affected (Fig. 1a, Table 2). The volume of the fibrosarcoma in dog #3 was decreased at the end of the pardaxin treatment period (Fig. 1b, Table 2). Dog patient #4 bore two squamous cell carcinomas; while the volume of one tumor (B) was no different before and after pardaxin treatment, the volume of the second tumor (A) was decreased (Fig. 1c, Table 2). Dogs #9 and 11 possessed malignant mast cell tumors; the volume of one of the tumors (B) in dog #9 was decreased at the end of the treatment period, while the volumes of the second tumor (A) in dog #9 and the tumor in dog #11 were unaffected (Fig. 1d, Table 2). The volume of the sarcoma in dog #12 was also unaffected by pardaxin treatment (Fig. 1e, Table 2).

**Table 2:** Table showing pardaxin intratumoral injection dose, injection frequency, tumor localization, tumor area before and after pardaxin administration, and the ratio of dose to superficial tumor area for each dog.

Pat no.	Dose per day	Injection site(s)	Tumor localization	Dose (mg/cm^2^)	Pre-treatment tumor area (cm^2^)	Post-treatment tumor area (cm^2^)
1	1 mg/ml ; 1 ml/day	2	A: right chest B: near right groin	A :0.124 mg/cm^2^ B: 0.090 mg/cm^2^	A: 8.050 cm^2^ B: 11.000 cm^2^	A:7.500 cm^2^ B: 13.530 cm^2^
2	4 mg/ml ; 1 ml/day	1	Right belly near shoulder	0.024 mg/cm^2^	160.095cm^2^	44.800 cm^2^
3	2 mg/ml ; 1 ml/day	1	Left front leg elbow	0.010 mg/cm^2^	182 .000cm^2^	142.800 cm^2^
4	4 mg/ml ; 1ml/day	2	A: belly near shoulder B: right front shoulder	A: 0.060 mg/cm^2^ B: 0.286 mg/cm^2^	A: 66.240 cm^2^ B: 13.940 cm^2^	A: 16.705 cm^2^ B: 13.250 cm^2^
5	4 mg/ml ; 1 ml/day	2	A: last right side breast B: last left side breast	A: 0.188 mg/cm^2^ B: 0.264 mg/cm^2^	A: 21.250 cm^2^ B :15.12 0cm^2^	A: 28.520 cm^2^ B: 18.480 cm^2^
6	10 mg/ml ; 2ml/day	1	In the 12 o'clock direction of anus	5.050 mg/cm^2^	3.960cm^2^	0.000 cm^2^
7	10 mg/ml ; 4 ml/day	1	Left front shoulder	4.629 mg/cm^2^	8.640 cm^2^	10.080cm^2^
8	10 mg/ml ; 1ml/day	1	A: left side first breast B: left side second breast C: right side first breast D: right side second breast	A: 0.512mg/cm^2^ B: 0.292mg/cm^2^ C: 4.347 mg/cm^2^ D: 8.695mg/cm^2^	A: 19.500 cm^2^ B: 34.200 cm^2^ C: 2.300 cm^2^ D: 1.150 cm^2^	A: N/A (deceased) B: N/A (deceased) C: N/A (deceased) D: N/A (deceased)
9	10 mg/ml ; 2 ml/day	2	A: right leg B: right leg	A: 4.166mg/cm^2^ B: 3.246 mg/cm^2^	A: 4.800 cm^2^ B: 6.160 cm^2^	A: 6.120 cm^2^ B: 2.250 cm^2^
10	10 mg/ml ; 2 ml/day	1	Left neck	4.545 mg/cm^2^	4.400 cm^2^	3.780 cm^2^
11	10 mg/ml ; 2 ml/day	1	Right leg	16.666 mg/cm^2^	1.200 cm^2^	1.530 cm^2^
12	10 mg/ml ; 4 ml/day	4	Left leg near shoulder	4.504 mg/cm^2^	8.880 cm^2^	15.390 cm^2^
13	10 mg/ml ; 0.5 ml/day	1	In the 12 o'clock direction of anus	3.918 mg/cm^2^	1.276 cm^2^	0.000 cm^2^
14	10 mg/ml ; 0.5 ml/day	1	A: upper left direction of anus B: upper right direction of anus	A: 2.463 mg/cm^2^ B: 1.666 mg/cm^2^	A: 2.030 cm^2^ B: 3.000 cm^2^	A: 0.000 cm^2^ B: 0.000 cm^2^

**Figure 2:**
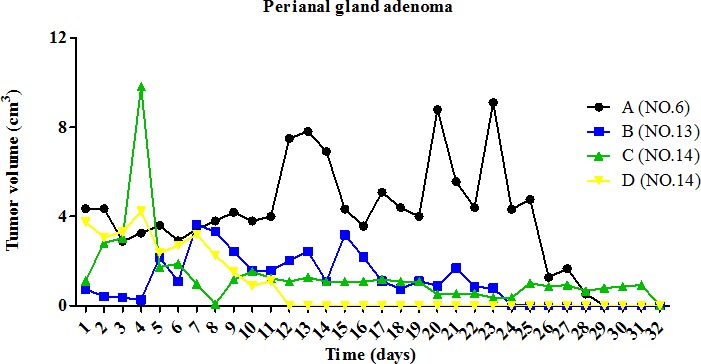
Change in tumor volume during treatment with pardaxin Four perianal gland adenomas (of dogs #6, #13, and #14) were monitored for 28 days. The first day of pardaxin administration is labeled as day one. Each tumor of each dog was injected with a different concentration of pardaxin, and results are plotted using different colors. The tumor volumes (cm^3^) of individual tumors are shown. Descriptions of tumor features and pardaxin treatment regimens are provided in Tables 1 and 2.

**Figure 3:**
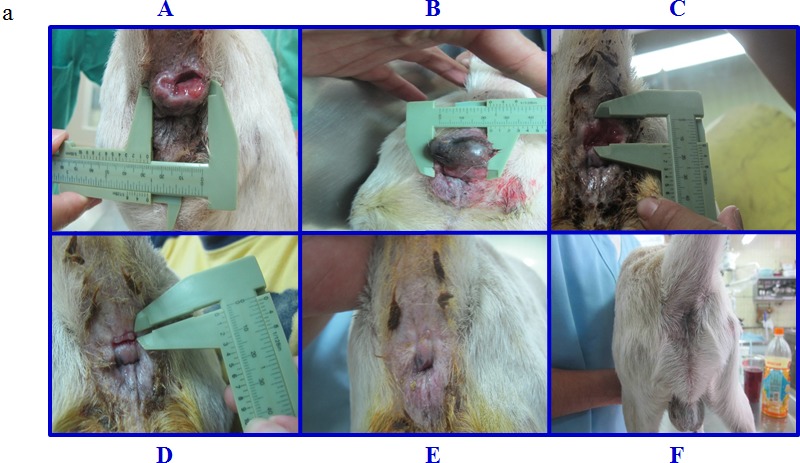

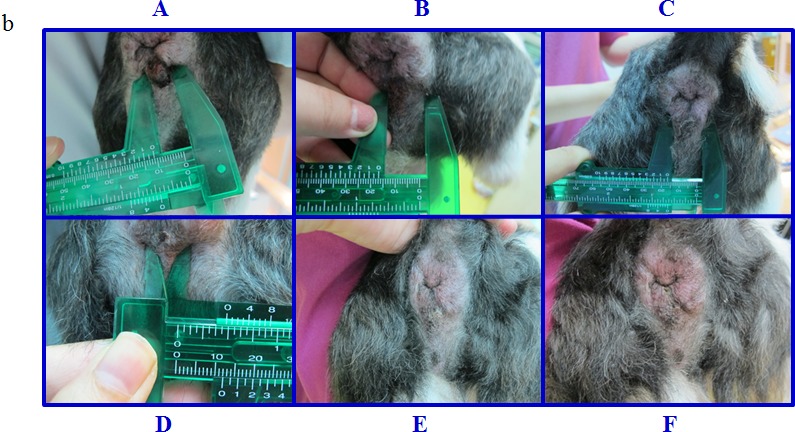

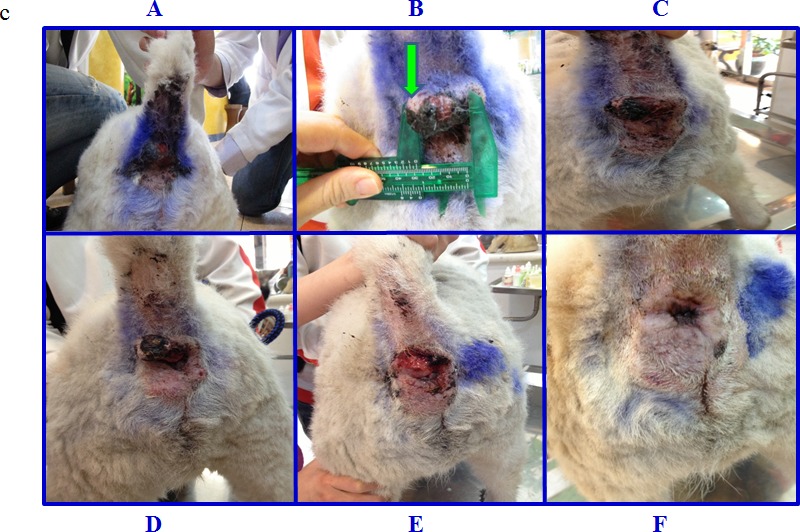

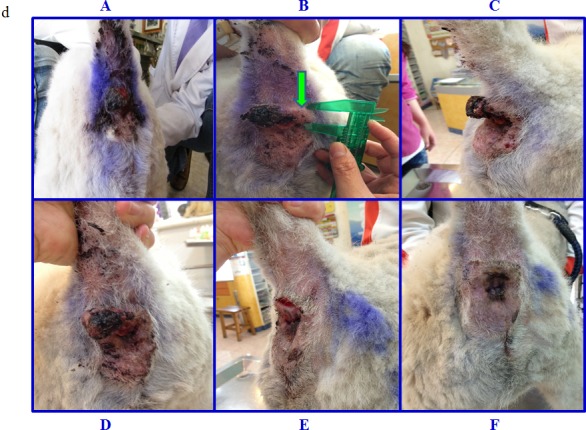
Morphological changes in the perianal gland adenoma of dog #6 during treatment with pardaxin for 28 days (a) (A) Lesions were ulcerated and protruding, due to the masses (arrow) in the 12 o'clock direction from the anus at day 0 (before pardaxin treatment). (B) The 17^th^ day after pardaxin treatment. The perianal gland adenoma exhibits cyanosis and appears black. (C) The 26^th^ day after pardaxin treatment. The perianal gland adenoma had burst open, leaving a hollow aperture. (D) The 33^rd^ day after pardaxin treatment. The wound had begun to heal. (E) The 37^th^ and (F) 121^st^ day after pardaxin treatment. Pardaxin treatment was stopped on the 29^th^ day. (b) Morphological changes in the perianal gland adenoma of dog #13 during treatment with pardaxin for 28 days. (A) Prior to treatment on day 0. (B) The 7^th^, (C) 14^th^, (D) 21^st^, (E) 24^th^, and (F) 28^th^ day of pardaxin treatment. (c) Morphological changes in the perianal gland adenoma to the upper left of the anus of dog #14 during treatment with pardaxin for 38 days. (A) Prior to treatment on day 0. (B) The 3^rd^, (C) 11^th^, (D) 17^th^, (E) 38^th^, and (F) 47^th^ day of pardaxin treatment. The green arrow indicates the location of the perianal gland adenoma. (d) Morphological changes in the perianal gland adenoma to the upper right of the anus of dog #14 during treatment with pardaxin for 31 days. (A) Prior to treatment on day 0. (B) The 7^th^, (C) 17^th^, (D) 21^st^, (E) 38^th^, and (F) 43^rd^ day of pardaxin treatment. The green arrow indicates the location of the perianal gland adenoma.

Dog patient #6, an 8-year-old, intact, mixed breed male, was observed to bear an intradermal nodule around the anus (3.960 cm^2^) prior to pardaxin treatment. After 28 days of pardaxin treatment, the perianal gland adenoma had disappeared (Fig 2). On physical examination, the lesion of the perianal area was found to be ulcerated (Fig. 3a). The perianal gland adenoma was surrounded by fibrous capsules and stroma. The dog received a single daily intratumoral injection of 10 mg/ml pardaxin (2 ml/day; pardaxin was dissolved in PBS). At day 19, a significant reduction in perianal gland adenoma volume was observed, and the adenoma mass ultimately burst (Fig. 3a (C, D)). The last day of pardaxin treatment was day 28. The wound remained under observation, and was found to have healed completely by the 121^st^ day (Fig. 3a). Thus, pardaxin treatment resulted in complete regression of tumor tissue of perianal gland adenoma. However, treatment with pardaxin induced rapid adenoma cell lysis, which manifested as a visible necrotic adenoma at 28 days after the first intratumoral injection.

Dog patients #13 and #14 received a single daily intratumoral injection of 10 mg/ml pardaxin (0.5 ml/day). In dog #13, pardaxin treatment resulted in a significant reduction in perianal gland adenoma growth, with the volume decreasing from 1.276 cm^2^ (pre-treatment) to 0.000 cm^2^ (post-treatment) (Table 2, Fig. 3b). No obvious necrotic adenoma areas were observed during the treatment period in dog #13 (Fig. 3b). Dog patient #14 possessed two perianal gland adenomas (Fig. 3c, 3d). Both perianal gland adenomas exhibited a decrease in volume; the first tumor decreased from 2.030 cm^2^ (pre-treatment) to 0.000 cm^2^ (38 days post-treatment; Fig. 3c), while the second decreased from 3.000 cm^2^ (pre-treatment) to 0.000 cm^2^ (31 days post-treatment; Fig. 3d) (Table 2).

**Figure 4:**
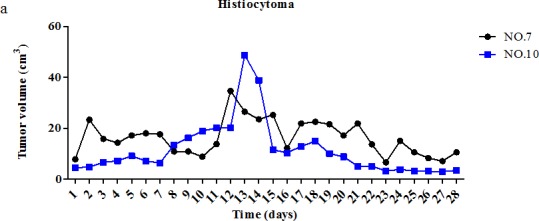

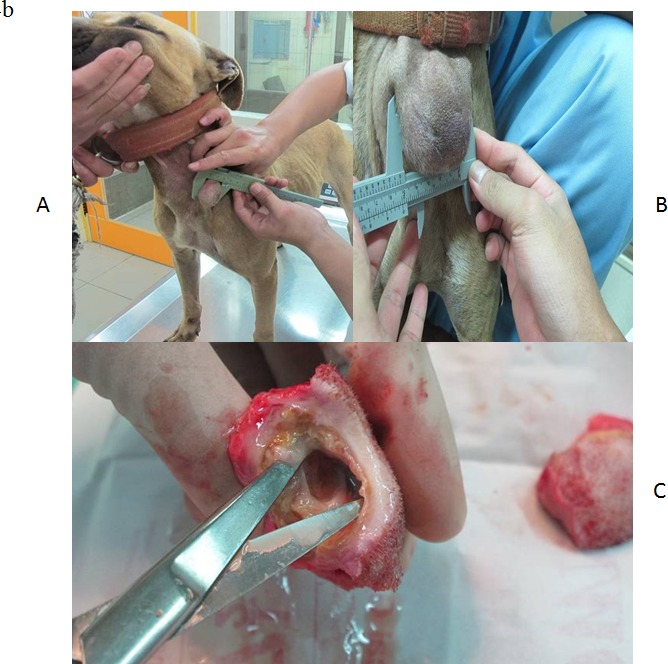
Change in tumor volume during treatment with pardaxin (a) Two histiocytoma tumors (of dogs #7 and #10) were monitored for 28 days. (b) (A) Prior to treatment on day 0 in dog #10. (B) Histiocytoma tumor after 19 days of pardaxin treatment. (C) Surgical excision of the tumor mass after 28 days of treatment with pardaxin; pus can be seen in the capsule, and there was no substantial tumor mass. The mass was hollow with extensive areas of necrosis after 28 days of daily intra-tumoral administration of pardaxin.

Dog patients #7 and #10 bore histiocytomas, which were not substantially affected by pardaxin treatment (Fig. 4a, Table 2). Dog #10, a 2-year-old American Pit Bull Terrier, presented with a histiocytoma in the left neck (4.400 cm^2^) (Fig. 4b). The dog received a single daily intratumoral injection of 10 mg/ml pardaxin (2 ml/day) dissolved in PBS. At day 19, a significant increase in tumor volume was observed, and the tumor mass felt very soft to the touch (Fig. 4b). Pardaxin treatment was halted at day 28. Subsequently, the tumor surface exhibited a small area of cyanosis (Fig. 4b). Surgical excision of the mass after the 28 days of pardaxin treatment revealed pus in the capsule and no significant tumor mass (Fig. 4b). The mass was hollow, with extensive areas of necrosis.

## DISCUSSION

In the current study, we used pardaxin to treat 14 dogs (belonging to private owners) with different tumor types; this investigation enabled us to identify pardaxin as a potent growth inhibitor of canine perianal gland adenoma *in vivo*. In recent years, antimicrobial peptides have garnered attention for their therapeutic potential against cancer [29, 30]. Our group has previously shown that intratumoral injection of antimicrobial peptides, such as pardaxin, can inhibit growth of syngeneic fibrosarcoma in a mouse model, without apparent toxic effects [10]. In this study, daily doses of pardaxin, ranging from 1 to 40 mg per dog, did not induce apparent drug-associated toxicity or abnormal blood biochemical parameters in tumor-bearing dogs. Moreover, pardaxin treatment did not significantly affect hematological properties, immune-related protein secretion, or biochemical parameters (Supplementary Table 1; Supplementary Figure 1). The dose-dependent cytotoxic effect of pardaxin was previously demonstrated *in vitro* (concentration range of 9 to 17 μg pardaxin/ml), by subjecting MN-11 cells to viability assays [10]. In the course of the current study, dog #8 died due to multiple organ dysfunction syndrome, possibly due to metastasis of the cancer. However, the owner did not consent to an autopsy, so we were unable to investigate the possibility of systemic toxicity. With the exception of dog #8, none of the dogs showed any signs of discomfort, pain, or toxicity after the injection of pardaxin.

To assess the effect of pardaxin on solid tumors *in vivo*, we injected the peptide locally into various tumor types in different dogs. A reduction in tumor growth was observed for all perianal gland adenomas, one squamous cell carcinoma, one fibrosarcoma, and one malignant mast cell tumor. Areas injected with pardaxin showed ulceration (Fig 3a and Fig 4b). Such ulceration in the lesion region was possibly a consequence of the pressure produced by the space-occupying mass or induced by cell necrosis. Induction of necrosis has been a consistent finding in previous studies of the effects of antimicrobial peptide on cancer, including studies of animals with solid tumors [12, 31]. Our earlier findings suggest pardaxin triggers apoptotic signaling pathways in human cervical carcinoma HeLa cells [27], consistent with findings that other antimicrobial peptides induce caspase-dependent apoptosis or programmed cell death of cancer cells via both mitochondrial and death receptor pathways [32, 33]. However, our observation of vacuolation at injected tumor sites in dogs suggests that pardaxin may interact with negatively-charged plasma membranes, causing their disintegration [34, 35]. In addition, pardaxin may be targeted to the endoplasmic reticulum, and mediate cell death by activating c-FOS [28]. However, it remains unknown whether differing doses of pardaxin, or differences in tumor or size, influence the cell lysis, pro-apoptotic, and/or anti-angiogenic effects of pardaxin.

Several earlier studies suggested that pardaxin exerts potent anti-tumor activity in cancer cell lines [10, 20, 27, 28], although the *in vivo* effects in dog, and the underlying mechanisms, remain unclear. Recent studies suggested that pardaxin induces apoptosis by triggering caspase-dependent and ROS-mediated apoptosis in human fibrosarcoma HT-1080 cells [20]. In the present study, pardaxin was shown to exert concentration- and time-dependent growth inhibition effects on canine perianal gland adenoma. The apoptotic effects of pardaxin were also found to be associated with TNF-α and IL-1β secretion level. Previous studies showed that TNF-α can sensitize MDA-MB-231 cells to Withaferin A and Celastrol, leading to apoptosis [36]. TNF-α induces apoptosis through its interactions with cell surface receptors, such as TNF-α receptor (TNFR) 1 and 2 (extrinsic pathway), and via mitochondrial dysfunction (intrinsic pathway) [37, 38]. It has also been reported that IL-1β induces the apoptosis of cells of the glioblastoma cell line GL15 by causing an imbalance between the MAPK and SAPK pathways [39]. Here, we show that the secretion of TNF-α and IL-1β by tumor-bearing dogs is gradually decreased after administration of pardaxin, implying that pardaxin may induce tumor cell apoptosis at dog tumor sites (Supplementary Figure 1).

This study was a dose-escalating trial; therefore, many dogs received low dosages of pardaxin. Furthermore, most of the dogs had been pre-treated with other drugs, and suffered from an advanced disease state. Therefore, the lack of an effect of pardaxin on the growth of certain tumors may be due to the dog's health. Partial remission in tumors that are difficult to treat, such as anaplastic mammary carcinoma, was not observed following pardaxin treatment. However, in general, perianal gland adenoma appeared to respond well to treatment with pardaxin. Most perianal gland swellings are focal hyperplasia of the benign proliferation form (adenoma); their counterparts (adenocarcinoma), on the other hand, are rare [40]. Most adenoma is effectively treated with cyclosporine, castration, and tumor removal with surgery [40, 41]. Estrogen treatment is another option for perianal gland adenoma when the owners decline castration. However, estrogen may have serious side effects, such as marrow suppression and fatal anaplastic anemia. Therefore, pardaxin may be considered as a possible candidate to replace estrogen for treatment of perianal gland adenomas in dogs.

From our biochemical analysis of the blood, it follows that treatment of dogs with pardaxin under physiological conditions is conducive to the local release of pardaxin with gradual internalization at the tumor sites; critically, slow internalization and intracellular release augments drug bioavailability. These properties lead to improved therapeutic responses, while avoiding excessive toxicity. Any systemic release of antimicrobial peptide in an intratumor space would be far less harmful than the use of organic compounds. In fact, preclinical toxicology studies in rodents and dogs have shown high tolerability to antimicrobial peptides. For example, the biochemical parameters measured here (Supplementary Table 1) exhibited no significant difference before and after treatment. In dogs, pardaxin did not significantly affect any measured biochemical parameters, including packed cell volume (P.C.V), red blood cell count, hemoglobin, mean cell volume (MCV), mean cell hemoglobin (MCH), mean corpuscular hemoglobin concentration (MCHC), leukocyte number, lymphocyte number, monocyte number, thrombocyte number, plasma, fibrinogen, total protein, T-bilirubin, AST, ALT, albumin, cholesterol, triglyceride, creatinine, blood urea nitrogen (BUN), or uric acid; this is indicative of high tolerability, and little/no toxicity to dog normal tissues. Analyses of enzymological and biochemical profiles of blood are widely used as indicators of the functional status of animal health. Variations in liver enzyme activities (AST, ALT, and T-bilirubin) imply liver dysfunction. An elevation in liver enzymes and/or certain chemicals (bilirubin and creatinine) usually indicates inflammation or damage to cells in the liver. As these liver markers were unaffected/decreased by pardaxin, it appears that treatment at the doses tested did not induce oxidative stress or hepatotoxicity. The biochemical findings were similar to those observed in mouse [10].

To the best of our knowledge, our report is the first to describe the use of an antimicrobial peptide as an anti-tumor agent in dog. We observed clear benefits of antimicrobial peptide therapy in dogs for which alternative treatment options, such as castration and tumor removal by surgery, were not available. In future studies, we may assess the effect(s) of combination(s) of pardaxin with homing peptide and nanoparticles.

## CONCLUSIONS

1. Pardaxin treatment has no visible side effects or overall toxicity on tumor-bearing dogs.
2. Pardaxin exhibits anti-tumor activity in tumor-bearing dogs, which is particularly potent against perianal gland adenoma, in which it leads to tumor shrinkage and loss.
3. Together with our previous results on the anti-tumor functions of pardaxin in allogeneic tumors in mice and an all-inclusive set of pre-clinical results, these experimental findings lend further support for future clinical development of pardaxin as a veterinary drug, and possible future suitability for human solid tumor pre-clinical trials.

## MATERIALS AND METHODS

### Materials

Pardaxin(GFFALIPKIISSPLFKTLLSAVGSALSSSGGQE) was synthesized and purified to a grade of >95% by GL Biochemistry (Shanghai, China). Synthetic peptides were dissolved in sterile deionized water or PBS buffer for the experiments.

### Clinical trial eligibility

All animal studies were performed in accordance with protocols approved by the Animal Care and Use Committee of National Pingtung University of Science and Technology. Dogs presented to the animal hospital of National Pingtung University of Science and Technology with histologically-, cytologically-, or morphologically-confirmed neoplastic disease were candidates for enrollment. Written informed consent from the owners of each dog was obtained prior to study enrollment. Surgery, chemotherapy, or other standard therapies were presented as options to owners of dogs with mammary gland tumors, perianal gland adenomas, sarcomas, or other solid tumors. Dogs for which conventional therapy had failed, dogs with owners who wished to try pardaxin treatment, and dogs with owners who declined offers of standard therapy were eligible for enrollment into this study.

### Study design and pardaxin peptide administration

A total of 14 privately-owned dogs with spontaneous tumors were enrolled in this study for open-label assessment of the safety and biological activity of pardaxin. Assessment of clinical toxicity and tumor response was performed every day. The dogs lived in the animal hospital, National Pingtung University of Science and Technology, for the duration of the study. Detailed information on dog breed, sex, age, and tumor type are provided in Table 1. Due to the different breeds of dogs included in this study, the body weight varied in the range 3~35 kg, with a mean of 17.78 kg. Dogs treated with pardaxin were subjected to routine blood biochemical parameter checks every 7 days, to evaluate hematological and biochemical toxicity. Pardaxin (0.5 ml to 4 ml) was injected into intratumoral sites at an initial dose of 1 mg/day, 2 mg/day, 4 mg/day, 5 mg/day, 10 mg/day, 20 mg/day, or 40 mg/day. Dog patients #1, #4, #5, and #9 were injected at two sites in the tumor surface. Circles were painted on the tumor, and separated into four quadrants. Pardaxin was injected into the fourth quadrant. Dog #12 was injected at four sites in the tumor surface area. Dogs #2, #3, #6, #7, #8, #10, #11, #13, and #14 were injected with pardaxin at one site in the tumor surface. Tumor size (in three dimensions) was measured every day during the treatment period using calipers. The first day before administration of pardaxin was designated as day 0, and treatment was continued until day 7, 12, 19, or 28. The two tumors of dog #14 were designated as A and B; injection of A was continued for 38 days and injection of B for 31 days. The treatment period duration was dependent on the treatment methods used for different dogs or tumor type (as shown in Table 2). The relationship between peptide dosage and tumor superficial area was defined as mg/cm^2^. Area was calculated as the product of tumor width and tumor region length.

### Blood collection and processing

Blood samples were collected from veins on the 1^st^, 7^th^, 14^th^, 21^st^, and 28^th^ day during treatment; samples were placed into EDTA tubes and centrifuged at 3000 × g for 10 minutes. Plasma was then collected for analysis of the biochemical parameters listed in Supplementary Table 1. Immune-related proteins (TNF-α, IL-1β, and MIP-1α) were detected by ELISA. Ethical approval was obtained from the Animal Care and Ethics Committee, National Pingtung University of Science and Technology.

### *In vivo* anti-tumor efficacy of pardaxin in tumor-bearing dog

Tumor size was calculated every day followed the formula: volume = length × width^2^ × 0.5 [28, 42]. The first day of administration was set at day 1.

## SUPPLEMENTARY MATERIAL, FIGURES AND TABLES


