# Supplementary material for: The antimicrobial peptide pardaxin exerts potent anti-tumor activity against canine perianal gland adenoma
Source: Oncotarget. 2014 Dec 10;6(4):2290–301. doi: 10.18632/oncotarget.2959 (PMC4385852; doi:10.18632/oncotarget.2959)
Supplement: Supplementary file 1 [file oncotarget-06-2290-s001.pdf]

The antimicrobial peptide pardaxin exerts potent anti-tumor activity against canine perianal gland adenoma

Supplementary Material

Supplementary Table 1: Biochemical analysis of the blood of the indicated dogs treated with pardaxin. Sera were collected before treatment (day 0) and on days 7, 14, 21, and 28 after primary injection. Sera were collected from dog #8 only on day 0 (pre-treatment). Biochemical parameters analyzed include packed cell volume (P.C.V), red blood cell count, hemoglobin, mean cell volume (MCV), mean cell hemoglobin (MCH), mean corpuscular hemoglobin concentration (MCHC), leukocyte number, lymphocyte number, monocyte number, thrombocyte number, plasma, fibrinogen, total protein, T-bilirubin, AST, ALT, albumin, cholesterol, triglyceride, creatinine, blood urea nitrogen (BUN), and uric acid.

	NO.1			NO.3					NO.4			NO.5		
Blood analysis	0 days	7 days	14 days	0 days	7 days	14 days	21 days	28 days	0 days	7 days	14 days	0 days	7 days	14 days
P.C.V	46.3/48	43.8/48	46/45	43.6/39	37.7/38	25.3/25	38.1/35	42.8/40	47.5/48	47.5/43	35.2/36	40.8/40	40.8/37	39.8/40
Red blood cell (1x10^6/ul)	7.09	6.66	7.12	6.38	5.41	3.64	5.49	6.42	7.28	7.63	5.49	6.18	6.19	6.13
Hemoglobin (g/dl)	16.4	15.9	16.8	13.8	13.4	8.8	13	14.3	16.5	16	12.3	14.1	13.8	13.6
MCV (fl)	65.3	65.3	65.3	65.3	65.3	65.3	65.3	65.3	65.3	65.3	65.3	65.3	65.3	65.3
MCH (pg)	23.1	23.1	23.1	23.1	23.1	23.1	23.1	23.1	23.1	23.1	23.1	23.1	23.1	23.1
MCHC (g/dl)	35.4	36.3	36.5	31.7	35.5	34.8	34.1	33.4	34.7	33.9	34.9	34.6	33.8	34.2

Total (leukocytes/ul)	19100	25900	16600	19500	13800	6200	15400	23600	12600	11500	13100	10100	11000	13300
Segments (%)	80	74	83	75	88	83	76	95	90	87	85	88	81	86
Lymphocytes	8	21	11	19	2	15	10	3	1	3	3	7	10	7
Monocytes	7	3	4	4	10		9	1	8	10	11	4	7	5
Thrombocytes (1x10 ^{3}/ul)	285	290	339	72	239	53	223	219	113	151	109	457	434	453
Plasma	Clear	Clear	slight hemolysis	clear	clear	clear	clear	clear	clear	clear	clear	clear	clear	slight hemolysis
Fibrinogen (g/dl)	0.6	0.4	0.4	0	0.2	0.2	0.2	0.6	0.6	0	0.6	0.2	0.8	ND
Total protein (g/dl)	6.6/6.0	6.4/6.0	7/6.6	8.6/8.6	8.4/8.2	8.2/8.2	8.6/8.4	9/8.4	7.4/6.8	6.4/6	5.8/5.2	9.2/9	9.6/8.8	9/8.8
T.Bilirubin (mg/dl)	0.4	0.3	0.5	0.4	0.3	0.4	0.6	0.4	0.5	0.6	0.6	0.5	0.9	0.7
AST (U/L)	30	29	36	61	43	27	34	42	41	47	47	42	47	51
ALT (U/L)	38	39	45	40	34	31	24	22	98	95	83	51	39	32
Albumin (g/dl)	2.9	2.8	3.1	3.5	3.3	3.1	3.2	2.8	3.2	2.7	2.5	3.1	ND	ND
Cholesterol (mg/dl)	165	197	193	245	235	234	253	210	187	167	147	313	336	289
Triglyceride (mg/dl)	63	66	78	47	48	58	49	48	52	117	99	73	119	132
Creatinine (mg/dl)	0.6	0.4	0.7	0.4	0.4	0.4	0.4	0.4	0.4	0.4	0.3	0.3	0.4	0.3
BUN (mg/dl)	17.6	12.8	18.4	9.9	7.7	7.8	10.7	6.5	7.1	6.6	6.5	ND	15.7	7.6
Uric acid (mg/dl)	0.4	0.4	0.4	0.5	0.6	0.6	0.7	0.7	0.6	0.7	0.6	0.7	0.6	0.7

	NO.6					NO.7					NO.8	NO.9				
Blood analysis	0 day	7 day	14 day	21 day	28 day	0 day	7 day	14 day	21 day	28 day	0 day	0 day	7 day	14 day	21 day	28 day
P.C.V	39.2/32	35.6/35	37.3/34	35.3/27	34.6/36	50.9/52	43.7/44	44.6/48	36/38	41.5/44	39.9/38	37/42	38.3/39	37.3/36	35.4/37	32.7/34
red blood cell ($1x10^6$/ul)	6.48	5.76	5.88	5.84	5.63	7.73	6.62	6.73	5.48	6.29	6.83	6.51	6.28	6.12	5.56	5.48
hemoglobin (g/dl)	13.2	12.6	12.5	13	12.1	17.6	15.2	15.5	12.3	14	14.8	14.4	13	12.7	12.5	11.7
MCV (fl)	65.3	65.3	65.3	65.3	65.3	65.3	66	64.4	65.7	66	65.3	56.8	61	60.8	60.4	59.7
MCH (pg)	23.1	23.1	23.1	23.1	23.1	23.1	23	22.4	22.4	22.3	23.1	22.1	20.7	20.8	21.3	21.4
MCHC (g/dl)	33.7	35.4	33.5	36.8	35	34.6	34.8	34.8	34.2	33.7	37.1	38.9	33.9	34.1	35.3	35.8
total (leukocytes/ul)	11400	9700	8800	8200	8100	31100	21100	24400	41200	34300	35500	11600	20200	17900	10700	10100
segments (%)	79	84	70	67	83	86	76	76	84	80	88	92	88	92	60	78
Lymphocytes	3	7	9	16	5	4	6	8	6	8	1	5	7	3	18	16
Monocytes	7	7	11	5	4	8	17	16	9	7	1	3	5	5	18	5
thrombocytes ($1x10^3$/ul)	133	72	62	117	145	223	292	282	185	352	94	87	198	305	379	187
Plasma	clear	clear	clear	clear	clear	clear	clear	clear	clear	clear	clear	clear	clear	clear	clear	clear
fibrinogen (g/dl)	0	0.8	0.6	0.2	0.2	0.8	0.9	0	0.6	0.2	0.8	0.2	ND	0.2	0.2	ND
total protein (g/dl)	9.8/9.8	9.6/8.8	11.2/10.6	10.6/10.4	8.4/8.2	8.8/8	9.0/8.1	8.0/8.0	8.2/7.6	8.2/8.0	7.7/6.6	6.4/6.2	5.6/5.2	6.2/6.0	7.0/6.8	6.4/6.4
T.Bilirubin (mg/dl)	0.3	0.3	0.2	0.2	0.2	0.5	0.4	0.5	0.5	0.5	0.5	0.5	1.3	0.3	0.5	0.5
AST (U/L)	47	35	1.7	30	42	33	22	28	24	37	81	65	23	25	32	23
ALT (U/L)	60	52	60	73	51	30	27	17	15	11	23	31	29	29	26	28

Albumin (g/dl)	3.1	3.1	ND	ND	ND	3.1	3.4	3.3	2.4	2.6	3.1	3	2.7	2.8	2.7	26
Cholesterol (mg/dl)	222	266	334	220	235	182	237	290	176	279	204	159	107	143	127	180
Triglyceriide (mg/dl)	28	130	30	44	45	49	90	71	101	114	54	66	114	92	89	83
Creatinine (mg/dl)	0.3	0.2	0.3	0.3	0.4	0.6	0.3	0.4	0.1	0.4	0.3	0.9	1.2	1.4	1.1	1
BUN (mg/dl)	9.3	13.7	10	9	11.9	13.5	10.8	7.2	8.8	7.9	11.8	23.7	29.6	25.2	29.4	25.4
Uric acid (mg/dl)	0.6	0.9	0.6	0.6	0.5	0.7	0.5	0.7	0.5	0.7	0.5	0.7	0.5	0.5	0.6	0.5

	NO.10					NO.11					NO.12				
Blood analysis	0 day	7 day	14 day	21 day	28 day	0 day	7 day	14 day	21 day	28 day	0 day	7 day	14 day	21 day	28 day
P.C.V	38.2/38	37.9/35	26.2/25	38.8/40	32.9/29	38.3/41	45.3/38	43.4/43	43.4/39	44.2/41	36.9/35	39.8/36	44/45	37.6/39	41.2/40
red blood cell ($1x10^6$/ul)	5.79	5.64	3.93	5.72	4.83	6.27	7.49	7.06	7.25	7.47	5.5	6.12	6.7	5.65	6.19
hemoglobin (g/dl)	13.5	13	9	13.1	10.4	13.6	14.2	14.6	14.2	14.2	12.8	13.7	15.2	13.1	14.4
MCV (fl)	66	67.2	66.7	67.8	68.1	61.1	60.5	60.1	59.9	57.22	67.1	65	65.7	66.5	66.6
MCH (pg)	23.3	23	22.9	22.9	21.5	21.7	19	20.7	19.6	19	23.3	22.4	22.7	23.2	23.3
MCHC (g/dl)	35.3	34.3	34.4	33.8	31.6	35.5	31.3	34.4	32.7	32.1	34.7	34.4	34.5	34.8	35
total (leukocytes/ul)	8900	10200	7750	7700	7300	9800	8000	10600	6000	8600	10300	10300	14700	20000	14000
segments (%)	81	68	52	75	50	56	73	78	53	68	67	68	72	82	56
Lymphocytes	15	19	36	10	30	24	24	14	24	20	27	19	18	10	30
Monocytes	4	7	3	5	5	15	1	7	19	9	2	11	7	6	10
thrombocytes ($1x10^3$/ul)	117	128	132	187	140	254	255	238	348	308	60	156	150	126	201
Plasma	clear	clear	clear	clear	clear	clear	clear	clear	clear	clear	clear	clear	clear	clear	clear
fibrinogen (g/dl)	0.2	1	0	0.4	0.4	0.2	0.2	0	0.2	0.4	0.2	0.2	ND	0.2	0.1
total protein (g/dl)	7.0/6.8	8.0/7.0	7.0/7.0	8.4/8.0	7/6.6	6.8/6.6	6.4/6.2	7.4/7.4	7.4/7.2	7.0/6.6	7.8/7.6	7.4/7.2	7.8/7.8	7.0/6.8	7.6/7.2
T.Bilirubin (mg/dl)	0.4	0.8	0.8	0.1	0.2	0.2	0.3	0.2	0.2	0.2	0.3	0.2	0.1	0.2	0.2
AST (U/L)	41	14	29	29	25	23	43	24	21	23	30	75	102	24	20
ALT (U/L)	68	75	87	134	78	24	24	26	22	27	115	65	401	88	57
Albumin (g/dl)	3	2.6	1.5	3	14.3	3.1	3	3	3	2.7	2.7	ND	ND	ND	ND

Cholesterol (mg/dl)	108	118	124	115	ND	181	227	226	225	188	354	220	230	211	191
Triglyceriide (mg/dl)	36	93	75	77	ND	74	97	107	72	60	56	41	115	59	48
Creatinine (mg/dl)	0.7	0.8	0.9	0.7	0.9	1	1	1.2	0.8	1	0.5	0.5	0.5	0.6	0.5
BUN (mg/dl)	13	18.5	16.5	16.6	14.3	22.1	19.8	24.5	22	16.5	18.7	14.7	14.3	13.7	16.9
Uric acid (mg/dl)	0.4	0.4	0.5	0.4	ND	0.4	0.6	0.4	0.4	0.4	0.6	0.3	0.5	0.4	0.4

	NO.13					NO.14				
	0 day	7 day	14 day	21 day	28 day	0 day	7 day	14 day	21 day	28 day
GLU (mg/dl)	106	87	100	90	99	114	118	119	99	101
BUN (mg/dl)	12	21	20	23	30	9	9	5	12	12
CREA (mg/dl)	1.1	1	0.9	1.1	1	1.2	1.3	1.1	1.2	1.3
PHOS (mg/dl)	3.5	6	5.6	5.5	5.5	4.2	4.6	4.0	4.9	4.6
CA (mg/dl)	10.6	10.6	10.9	ND	10.6	10.3	10.3	10.4	10.1	9.9
TP (g/dl)	7.2	7.4	7.8	8.1	7.5	6.9	7.4	7.7	7.3	7.2
ALB (g/dl)	3	2.9	3.1	3	3	3.3	3.1	3.3	3.3	3.3
GLOB (g/dl)	4.2	4.5	4.7	5.1	4.5	3.6	4.3	4.4	4.0	3.9
ALT (U/L)	85	56	87	26	24	34	51	52	30	32
ALKP (U/L)	135	101	106	95	73	183	140	229	182	151
TBIL (mg/dl)	0.4	0.4	0.4	0.3	0.3	0.3	0.2	0.3	0.3	0.3
CHOL (mg/dl)	183	204	210	222	221	178	185	204	169	171
AMYL (U/L)	1040	1332	882	899	829	779	835	470	732	727
WBC ($1x10^9$/ l)	14.6		14	9.9	11.6	12.1	ND	14.4	13.3	10.6
LYM ($1x10^9$/ l)	3.8		2	1.3	3.3	ND	ND	ND	ND	ND
MID ($1x10^9$/ l)	2.5		1.4	1	1.9	ND	ND	ND	ND	ND
GRAN ($1x10^9$/ l)	8.3		10.6	7.6	6.4	ND	ND	ND	ND	ND
LYM (%)	25.9		14.2	13.7	28.3	ND	ND	ND	ND	ND
MID (%)	16.9		10	9.4	16.2	ND	ND	ND	ND	ND
GRA (%)	57.2		75.8	76.9	55.5	ND	ND	ND	ND	ND

HGB (g/dl)	15	15	19.5	14.2	16.3	69.6	16.5	14.6	15.1
HCT (%)	40.1	40	53.3	38.5	47.6	46.4	48.2	44.0	45.6
RBC ($1x10^{12}$/ l)	6.63	6.59	8.75	6.2	7.68	7.68	7.94	7.33	7.25
MCV (fl)	60.4	60.6	60.8	62	62.0	60.4	60.7	60.0	60.6
MCH (pg)	22.7	22.7	22.3	23	21.0	90.6	20.8	19.9	20.1
MCHC (g/dl)	37.6	37.5	36.6	37.1	34.3	ND	34.2	33.2	33.1
RDW (%)	20.3	20.4	21.1	20.3	ND	ND	ND	ND	ND
RDWa (fl)	43.3	43.3	45.8	44	ND	ND	ND	ND	ND
PLT ($1x10^{9}$/ l)	318	317	276	347	306	315	177	278	288
MPV (fl)	6.5	6.5	6.6	6.3	9.5	8.8	9.3	9.4	9.4

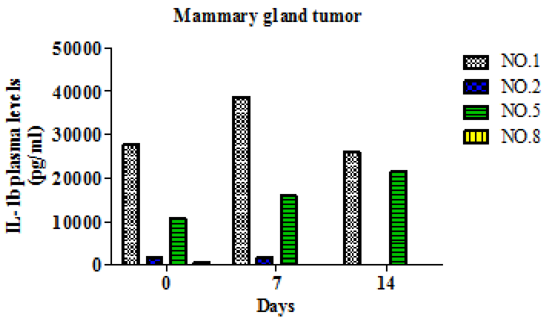
Mammary gland tumor
IL-1b plasma levels (pg/ml)
50000
40000
30000
20000
10000
0
0
7
14
Days
NO.1
NO.2
NO.5
NO.8

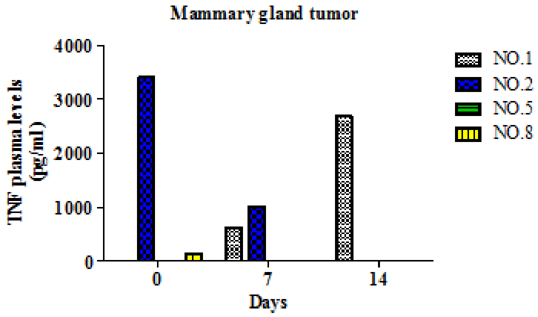
Mammary gland tumor
TNF plasma levels (pg/ml)
4000
3000
2000
1000
0
0
7
14
Days
NO.1
NO.2
NO.5
NO.8

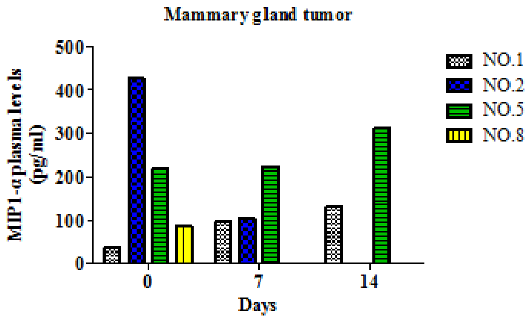
Mammary gland tumor
MIP1-α plasma levels (pg/ml)
500
400
300
200
100
0
0
7
14
Days
NO.1
NO.2
NO.5
NO.8

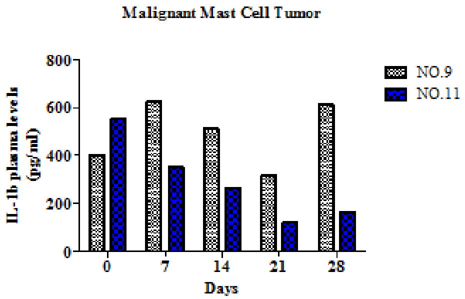
Malignant Mast Cell Tumor
IL-1b plasma levels (pg/ml)
800
600
400
200
0
0
7
14
21
28
Days
NO.9
NO.11

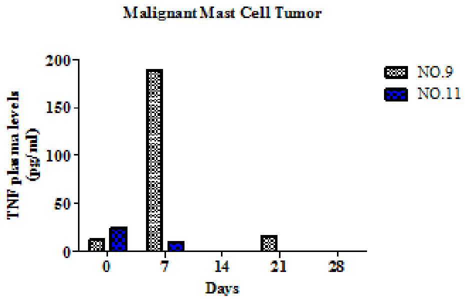
Malignant Mast Cell Tumor
TNF plasma levels (pg/ml)
200
150
100
50
0
0
7
14
21
28
Days
NO.9
NO.11

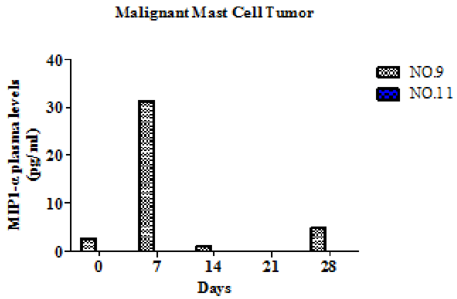
Malignant Mast Cell Tumor
MIP1-α plasma levels (pg/ml)
40
30
20
10
0
0
7
14
21
28
Days
NO.9
NO.11

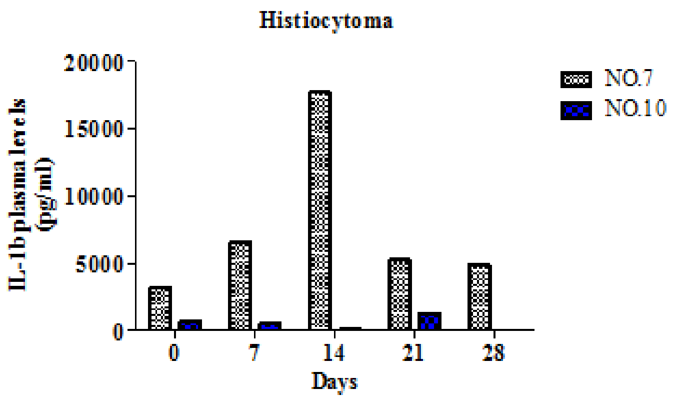
Histiocytoma
IL-1bplasma levels (pg/ml)
20000
15000
10000
5000
0
0
7
14
21
28
Days
NO.7
NO.10

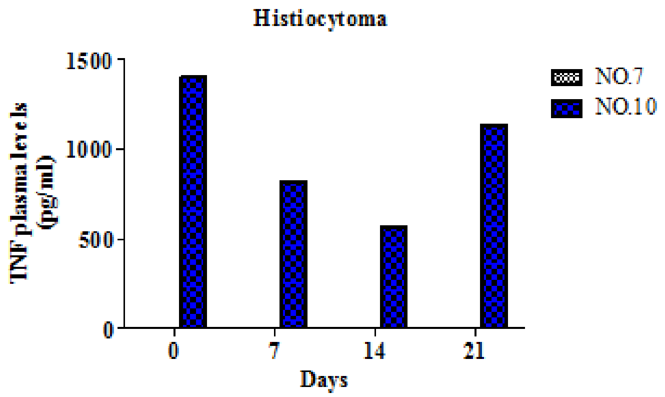
Histiocytoma
TNF plasma levels (pg/ml)
1500
1000
500
0
0
7
14
21
Days
NO.7
NO.10

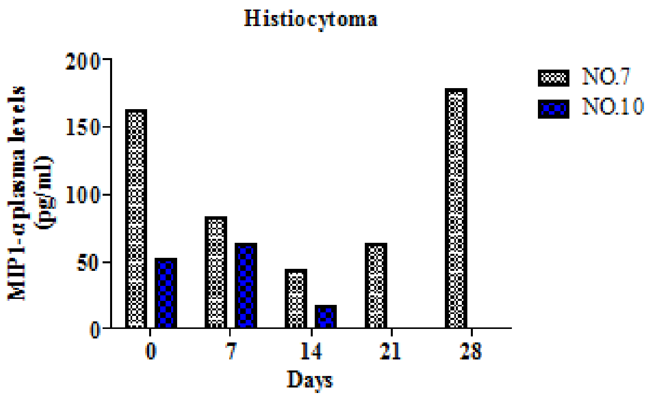
Histiocytoma
MIP1-α plasma levels (pg/ml)
200
150
100
50
0
0
7
14
21
28
Days
NO.7
NO.10

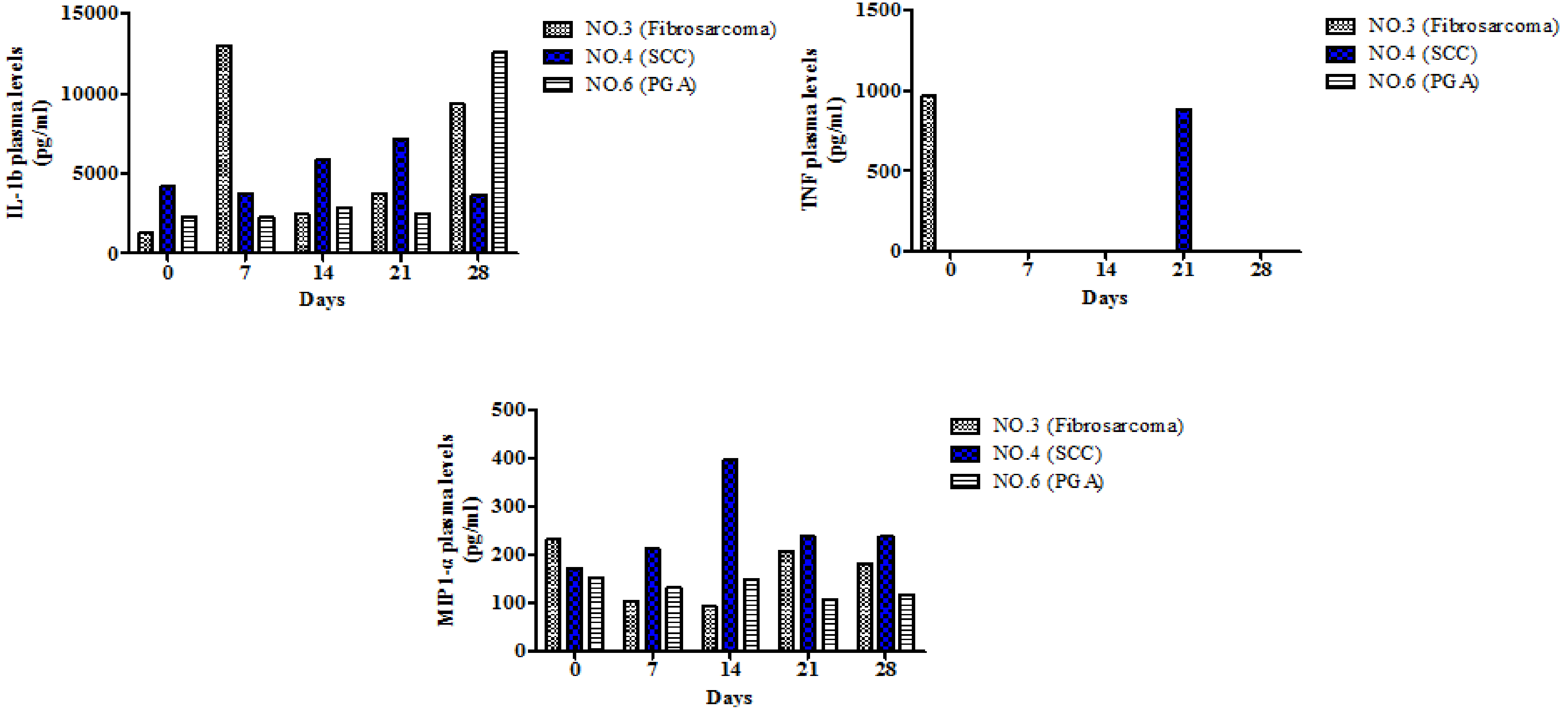

Supplementary Figure 1: Changes in serum secretion of macrophage inflammatory protein (MIP)-1, IL-1β, and tumor necrosis factor (TNF)-α at the indicated times after pardaxin treatment (day 0: pre-treatment). Dog numbers refer to the dogs described in Tables 1 and 2.
